# Immunopathogenesis of viral infections in neurological autoimmune disease

**DOI:** 10.1186/s12883-023-03239-x

**Published:** 2023-05-23

**Authors:** Mohammad Amin Habibi, Fatemeh Nezhad Shamohammadi, Taraneh Rajaei, Haideh Namdari, Mohammad Reza Pashaei, Hamid Farajifard, Sajjad Ahmadpour

**Affiliations:** 1 Multiple Sclerosis Research Center, Neuroscience Institut, Tehran University of Medical Sciences, Tehran, Iran; 2 Pediatric Cell and Gene Therapy Research Center, Gene, Cell and Tissue Research Institute , Tehran University of Medical Sciences, Tehran, Iran; 3 Department of Immunology, School of Medicine, Shiraz University of Medical Sciences, Shiraz, Iran; 4 Department of Microbiology and Immunology, Faculty of Veterinary Medicine, University of Tehran, Tehran, Iran; 5 Iranian Tissue Bank and Research Center, Imam Khomeini Hospital, Tehran University of Medical Science, Tehran, Iran; 6 Department of Internal Medicine, School of Medicine, Patient Safety Research Center, Clinical Research Institute, Urmia University of Medical Science, Urmia, Iran; 7 Patient Safety Research Center, Clinical Research Institute, Urmia University of Medical Sciences, Urmia, Iran

**Keywords:** Multiple sclerosis, Autoimmunity, Viral infections, Autoimmune reaction

## Abstract

Autoimmune diseases develop due to self-tolerance failure in recognizing self and non-self-antigens. Several factors play a role in inducing autoimmunity, including genetic and environmental elements. Several studies demonstrated the causative role of viruses; however, some studies showed the preventive effect of viruses in the development of autoimmunity. Neurological autoimmune diseases are classified based on the targets of autoantibodies, which target intracellular or extracellular antigens rather than neurons. Several theories have been hypothesized to explain the role of viruses in the pathogenesis of neuroinflammation and autoimmune diseases. This study reviewed the current data on the immunopathogenesis of viruses in autoimmunity of the nervous system.

## Introduction

The immune system can identify and remove invading pathogens and prevent infection. Self-tolerance is described as a condition of the immune system that is not reactive to the self-antigen. A self-tolerance process is carried out throughout the immune system development, categorized as either central or peripheral tolerance [1]. The breakdown of self-tolerance and an abnormal immune response to self-antigen could result in autoimmune disorders. Many factors, including genetics, age, and environmental factors, have been found to trigger inflammation and autoimmune reactions [1]; however, the exact etiology of several autoimmune diseases is still unknown. Viruses have long been regarded as an important environmental trigger for autoimmune diseases in genetically predisposed patients [2, 3]. They might activate some immunological responses through self-tolerance breakdown, which might overcome the immune regulating systems and induce autoimmune reactions. The most important known mechanisms in developing virus-induced autoimmunity are molecular mimicry between host self-antigens and microbial antigens, epitope spreading, bystander activation, and immortalizing infected B cells. Molecular mimicry plays a critical mechanism responsible for viruses-induced autoimmune disease. Conventionally, molecular mimicry applies to the similarity of antigens between viruses and self-antigens that can be recognized by immune systems and result in cross-reaction to self-antigens and viral antigens. The epitope spreading is another main mechanism responsible for the viruses-induced immune reaction in which viral infection results in more discharge of self-antigens and novel autoreactive cells that subsequently target spared self-antigens [4]. However, the exact contributing mechanism is poorly understood. In the recent COVID-19 pandemic, many COVID-19-associated autoimmune disorder cases have drawn particular attention to the neuropathogenesis of viral infection.

Given the increasing evidence suggesting the association between viral infection and autoimmune disorders, and controversial data on the role of viruses in dysregulation of the immune response, herein, we aim to review the current data on the immunopathogenesis of the common viruses in developing nervous system autoimmune disorders.

## Methods

PubMed/Medline electronic database was searched using the keywords “Guillain–Barre syndrome”, “Myasthenia Gravis”, “autoimmune disease”, “Multiple sclerosis”, “experimental autoimmune encephalitis”, “central nervous system”, “COVID-19”, “HSV-1”, “Influenza”, “Epstein barre virus”, “EBV”, “CMV”, “Cytomegalovirus”, “Zika Virus”, and “varicella-zoster virus”. We reviewed the English articles with full-text available between January 1, 2000, to March 1, 2022. A total of 1210 articles were retrieved, and 283 articles were included in this study. The inclusion criteria for this narrative review were studies on the role of selected viruses in developing the neurological autoimmune diseases.

### Autoimmune diseases of the nervous system

The incidence of autoimmune diseases is estimated to be more than 5 percent in the general population, with an increasing prevalence in recent years [5]. Up to date, About 80 autoimmune disorders have been identified, including nearly 30 neurological autoimmune diseases [6]. Neurological autoimmune diseases are classified based on the targets of autoantibodies, whereas autoantibodies target intracellular or extracellular antigens of the neurons [7]. Antibody-associated autoimmune diseases against intracellular antigens are often associated with underlying malignancy and are defined as paraneoplastic disorders [7]. Mis-response of the immune system to the ectopic neural antigens, which are aberrantly expressed in malignant cells, direct the hypothetical mechanism of these disorders. Previous studies have shown that autoantibodies against intracellular antigens were not in relation to the target antigens and, therefore, are not responsible for disease development. As a result, these patients have an inadequate response to the treatment. In this regard, some studies investigated the mechanism of autoimmune diseases with intracellular antigens revealing that CD8 + T cells penetrated the neurons and induced granzyme B and perforin production, ultimately leading to neural degeneration [8].

Autoimmune diseases associated with the autoantibodies against extracellular epitopes, including cell surface and synaptic antigens, are less related to underlying malignancy. Moreover, in contrast to paraneoplastic CNS disorders, they are characterized by good responsiveness to immunosuppression. There is also a more complicated autoantibody expression pattern, mainly exposed during synaptic fusion and reuptake [8].

### Viruses-induced neurological autoimmunity

Some theories have been postulated to explain the role of viruses in the pathogenesis of autoimmune diseases; however, the exact mechanism of viruses-induced neurological autoimmune disorders is still unknown. Altogether four main mechanisms were identified for viruses-induced neurological autoimmune disease: molecular mimicry, epitope spreading, bystander activation, and autoantibody production and immortalization of effector B-cells. Molecular mimicry is defined by similar antigens of self-epitopes and pathogen's antigens, resulting in a cross-reactive reaction of B and T cells to the self-antigen that caused autoimmune disease [9]. The innate immune system causes bystander activation. The immune reaction of the innate immune system provides a strong response against pathogens through the massive production of pro-inflammatory cytokines and chemokines. The overactivation of the immune system's exaggerated response against viruses caused a cytokine storm that initiated additional damage to the neurological tissues and produced more self-antigens. The novel self-antigens are further presented by antigen-presenting cells (APCs) to the autoreactive immune cells and thus trigger an in-process autoimmune reaction [10]. Epitope spreading is another possible mechanism involved in viral-induced neurological autoimmune diseases. Further self-antigens are presented upon ongoing damage to self-tissue and infliction caused by viruses, and other immune reactions were induced by autoreactive T cells to the novel self-antigens [11]. Self-antigen autoantibodies and immortalized effector B cells are caused by memory and affect B cells. Alongside the typical autoantibodies, patients with neurological autoimmune disease can be presented with some other autoantibodies in the nervous system tissues. Additionally, immune system memory cells stimulate effector B cells trained against self-antigens and cause continuous long-term autoantibody production against nervous system antigens [12, 13].

In this study, we sought to review the immunopathogenesis of viruses playing a role in the induction of autoimmunity of the nervous system, especially following the advent of the COVID-19 pandemic.

### Cytomegalovirus

Cytomegalovirus (CMV), the linear double-stranded DNA virus, is a member of the *Herpesviridae* family, introduced in 1904. The virus infects 60 − 100% of people in adulthood [14, 15]. The strong interaction between CMV and the immune system has highlighted its role in inducing autoimmune diseases. Increasing evidence has shown the association of CMV infection with rheumatologic diseases such as systemic lupus erythematosus (SLE), systemic sclerosis (SSc), rheumatoid arthritis (RA), and some neurological disorders. In contrast, there are reports revealing the protective impact of CMV in some autoimmune diseases such as celiac disease [16]. Moreover, there are controversial reports on the association between CMV and MS. While some reports have suggested the association between reactivation of CMV infection and the development or worsening of pre-existing MS, other studies have shown a negative correlation between the development of MS and CMV seropositivity [17].

Following viral penetration, pattern recognition receptors (PRRs) identify the pathogen-associated molecular pattern (PAMP) and the damage-associated molecular pattern (DAMP) of the virus, which induce the immune reaction to the virus [18]. Molecular mimicry is assumed as another responsible mechanism for CMV-induced autoimmunity. An animal model study demonstrated some degree of molecular mimicry between the myelin oligodendrocyte glycoprotein 35–55 (MOG35-55) and one of the CMV peptides [19]. Immunization of mice with MOG35-55 following murine CMV (mCMV) infection induced symptoms similar to the experimental autoimmune encephalomyelitis (EAE) and increased the influx of T cells (Th-1 and Th-17) into the CNS. In contrast, immunization with MOG35-55 without CMV infection was not able to induce EAE [20]. The cross-reactivity of MOG peptide and CMV peptide has also been proven in a non-human model of primate since MOG34–56 specific T cells responded to the human CMV major capsid protein (UL86; 981–1003) [21].

**Fig. 1:**
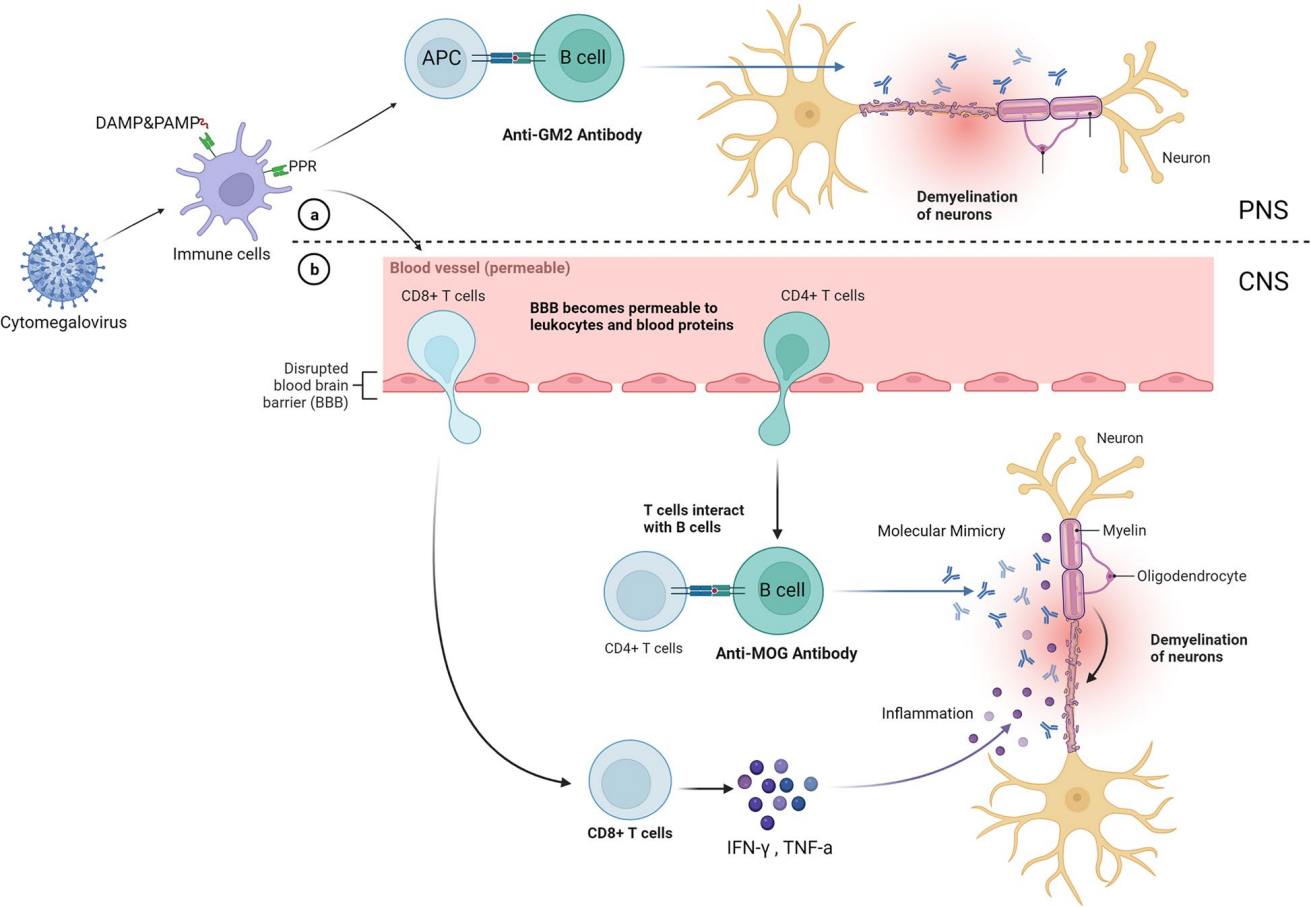
The mechanism of CMV-induced multiple sclerosis. Immune reaction against the CMV induces autoantibody productions. CMV infection is also associated with a T-cell influx to the CNS, resulting in inflammation. Created with BioRender.com

There is also evidence suggesting the association between CMV and CD4 + CD28null T cells. Human studies have shown a direct relationship between the titer of CMV seropositivity and the number of CD4 + CD28 null T cells [22]. In vitro studies have demonstrated that stimulation with CMV enhances the CD4 + CD28 null T cells population, which all contribute to aggravating the symptoms of EAE [22]. In addition, animal and human fetus studies have shown that CMV infection of the CNS induces CD8 + cells, interferon gamma (IFN-γ), and tumor necrosis factor α (TNFα) accumulation in the CNS, leading to CNS inflammation [19]. The mechanism of CMV-induced multiple sclerosis (MS) is given in Fig. 1.

### Zika virus

Zika virus is one of the members of the *Flaviviridae* family, with a single-stranded positive-sense RNA genome, which can participate in the development of some central and peripheral nervous system disorders such as Guillain-Barré syndrome (GBS), transverse myelitis (TM), and meningoencephalitis [23, 24]. Although it was shown that Zika virus infection can be presented wide range of neurological manifestations [25], few studies reported detection of Zika virus in patients with MS [26]. It is suggested that zika virus can induce autoimmune reaction against neural cells that can mimic the presentation of MS [27].

Despite the effect of Zika virus in neurodevelopmental process and congenital pathologies are well understood [4, 28], the impact of zika virus on neural process of adults are remained unknown and the mechanisms underlying ZIKV-induced neuropathogenesis are still poorly understood. It was shown that Zika virus can induce demyelinating process and also axonal injury of neuron related to the CNS that can underlie autoimmune disease of CNS [29]. However, murine studies have demonstrated that ZIKV can replicate and affect the CNS cells, stimulate the expression of inflammatory genes such as interleukin-1 (IL-1) and enhance the expression of NLR family pyrin domain containing 3 (NLRP3) and some other genes responsible for oxidative stress [30]. Other studies have shown that Toll-Like Receptor 3 (TLR3), a part of the innate immune system, is involved in the ZIKV-induced neuropathogenesis since inhibition of the TLR3 function reduces the viral replication and decreases the secretion of inflammatory mediators such as IFN-β and IL-6 from immune cells [31]. Notably, IFN-I pathways are activated during viral infection, allowing the expression of hundreds of elements involved in the IFN-stimulated response. ZIKV non-structural protein 5 (NS5) binds and destroys the Signal Transducer and Activator of Transcription 2 (STAT2) via proteasomal degradation, conferring viral resistance to IFN in cell cultures. Inhibition of IFN is the first step of ZIKV pathogenesis. Following IFN hampering, transmembrane proteins activated by IFN are decreased, resulting in elevation of the ZIKV-caused cell death [32]. It should be noted that following viral invasion, the retinoic acid-inducible gene 1 (RIG-I)-like receptors (RLRs) as viral RNA detectors are responsible for initiating the innate immune response, which the IFN-I mediates. While the ZIKV can inhibit the IFN-I, it can directly activate the RLRs inducing the production of proinflammatory cytokines. Subsequently, different subsets of CD4 + T cells will be activated against ZIKV, leading to its neuropathogenesis [32]. In addition to the production of inflammatory cytokines, higher levels of chemoattractive molecules produced by the ZIKV-infected blood–brain barrier (BBB) cells such as CCL5, CCL2, and CXCL10 increase the influx of immune cells into the CNS, which eventually leads to an inflammatory reaction in the CNS [33, 34]. Besides, ZIKV-induced CCL5 production could inversely affect the myelination process. Last but not least, ZIKV can affect other cells in the nervous system except for neurons. Some studies have shown that ZIKV induced morphological changes in astrocytes and fibroblasts, which might contribute to the neuropathogenesis of ZIKV [32].

### Varicella-zoster virus

Varicella-zoster virus (VZV), known to cause chickenpox infection in humans, is one of the most frequent viral infections, affecting about 95% of adults in developed countries. After the initial infection, it establishes latency in the dorsal ganglia of most healthy people, which then might reactivate in particular circumstances [35]. Some studies have confirmed a positive association between VZV infection and the risk of developing autoimmune diseases such as MS [36–38]. A survey of a large population of MS patients and healthy individuals demonstrated that antibodies against VZV and CMV were significantly higher in MS patients than in healthy individuals [39]. Furthermore, unlike healthy individuals, the presence of VZV particles has been established in the urine of MS patients [40]. Likewise, CNS examination of MS patients has shown a higher percentage of VZV particles in the MS patients' CNS than in healthy individuals [41]. Interestingly, several studies have revealed that the VZV viral load in the CNS and peripheral blood of MS patients in the relapse phase was significantly higher relative to the remission phase [42], highlighting the hypothesis that VZV might play a role in developing or exacerbating MS symptoms. However, others have failed to show the presence of VZV virions or DNA in the CSF in the acute plaques of MS patients, which calls into question the validity of this hypothesis.

Molecular mimicry, as an old hypothesis for explaining the possible role of viruses in inducing autoimmune diseases also has been suggested for the relationship between VZV and MS development. Degrees of molecular mimicry exist between VZV glycoprotein E and Heterogeneous Nuclear Ribonucleoprotein A1 (HNRNPA1), which is present in the nucleoplasm as it shares > 62% amino acid sequence similarity with the prion-like domain (PrLD) of HNRNPA1, signifying the reason behind autoantibodies against M9 and PrLD of HNRNPA1. HNRNPA1 mutation might stimulate the presence and enhancement of HNRNPA1 in the cytoplasm, along with the presentation of the protein by MHC-1, which all trigger a cascade of immune reactivation [43].

Bearing in mind all considerations, the evidence favors the contributing role of VZV in inducing MS. However, further studies with more rigorous methodologies are needed to support this hypothesis.

### Epstein–Barr virus

Epstein–Barr virus (EBV) is a ubiquitous member of the gamma-herpesvirus subfamily that is common in humans. The silent infection and the life-long persistence are the keys to the widespread infection of EBV in the human population. The virus is a linear DNA virus that encodes about 100 proteins and 44 micro RNAs (miRNAs). While many EBV miRNAs have no known function, there is evidence suggesting the role of viral miRNAs in innate immunity by regulating the inflammasome component NLRP3, the natural killer group 2D (NKG2D) ligand MICB, and the chemokine CXCL11 [44–46]**.**

Following initial infection, EBV crosses the Waldeyer’s ring to infect the naïve B cells. The activation of these naïve B cells to proliferating lymphoblasts is mediated via the Epstein-Barr nuclear antigen (EBNA). The activated lymphoblasts then migrate to germinal centers where they undergo a germinal center reaction to access their primary target (resting memory B cells) for latent persistence. The signals from the EBV encoded latent membrane proteins (LMPs) contribute to the survival of these infected lymphoblasts. It is noteworthy that given the type of latency, the virus expresses different sets of latent products including LMP1 and LMP2 [39, 40].

It is believed that EBV-associated pathologies result from the disruption of the virus-host immune system balance, and clinical manifestations of EBV infection emerge as a result of provoked immune response rather than EBV itself. In this regard, several studies have shown the immune response to EBV is disturbed in MS patients. *Sumaya *et al*.* were the first ones who described an increased frequency of antibodies to EBV in patients with MS compared to healthy controls. Since then, many studies have demonstrated increased antibodies against EBV antigens titer in MS patients [47–49]. Moreover, several studies have revealed evidence of EBV particles or EBV genomes in the brain tissue samples of MS patients [50]. Interestingly, in a recent issue of Science, *Bjornevik and Cortese *et al*.* utilized longitudinal evaluation of over 10 million adults between 1993 and 2013 to demonstrate the association between EBV infection and MS development. They showed a 32-fold increase in the risk of MS following EBV infection, but the risk was not increased after other viral infections. Moreover, neurofilament light chain levels were increased only after EBV seroconversion.

Notably, many hypotheses have been proposed to express the role of EBV in developing MS. Molecular mimicry has repeatedly been suggested as a potential pathogenic mechanism. The LMP1 mimics CD40 receptors, which play a role in B and T-cells interactions, and LMP2A mimics B-cell receptors. Moreover, an IL-10-like cytokine that EBV produces is crucial to B-cell activations [51]. In addition, patients with MS exhibit a more robust humoral response to EBNA-1, EBNA411–426, and EBNA1400–413, which can interact with some peptides of the myelin essential proteins as glial cell adhesion molecule (GlialCAM) [52, 53]. Likewise, elevated antibodies against the chloride-channel protein anoctamin 2 are seen in MS patients, which could cross-react with one of the EBNA1 peptides [54]. The molecular mimicry between EBV antigens and MS autoantigens has been confirmed in animal models. Namely, stimulation of mice with EBNA411–426 has been shown to increase the portion of T cells (IFN-γ producers) in response to MBP, leading to EAE [53].

Furthermore, it seems there is a relationship between EBV-infected B cells and the development of MS. Notably, EBV micro RNAs can protect EBV-infected B cells from CD8 + T cell response by reducing the EBV-specific CD8 + T cell proliferation and IFN-γ secretion [46]. Interestingly, it has been demonstrated that the number of T cells that recognize EBV-infected B cells decreases in MS subjects [55]. In addition, in contrast to healthy individuals, the latent-specific CD8 + T cells population is significantly greater than the lytic-specific CD8 + T cells [55, 56]. Moreover, IFN-γ secreted by CD8 + T cells hampers the EBV-infected B cell’s proliferation and decreases the function and number of EBV-specific CD8 + T-cells, which results in intact EBV-infected B cells [57]. Therefore, it can be concluded that in healthy people, EBV-specific CD8 + T cells with an appropriate ratio of the lytic and latent specific CD8 + T cells could kill the EBV-infected cells. However, the number of lytic-specific CD8 + T cells is insufficient in MS patients, limiting their ability to regulate the EBV infection effectively. Moreover, while the number of EBV-latent CD8 + T cells is significantly higher in MS patients, it is insufficient to prevent the growth of infected memory B cells. What is more, after a while, they show exhaustion which leads to more inefficiency [55].

**Fig. 2:**
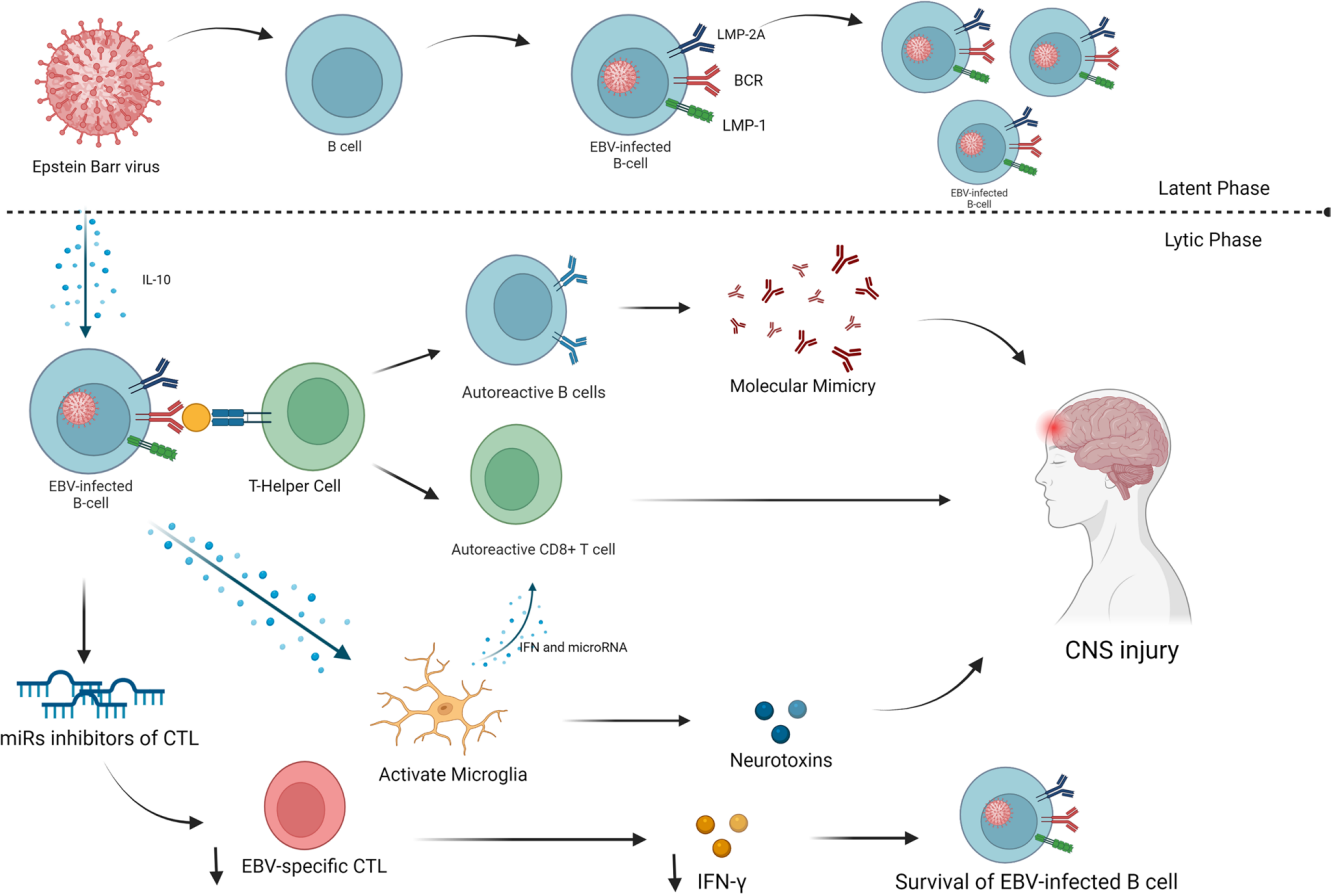
The mechanism of Epstein-Barr Virus-induced Nervous System damage. EBV life cycle has two phases, including latent phase which provided proliferated viruses without immune reactions. When the number of viruses increased, the immune system reacted against viruses. The EBV-infected B-cells activate which subsequently activate adaptive lymphocytes. The EBV-infected B-cells produce some microRNAs that decrease EBV-specific T-cells and IFN-γ production which resulted in sustained EBV-infected B-cells. Prolonged EBV infections cause changes in the neuroprotective state of microglia to the neurodegenerative state which include neurotoxin production and activation of immune cells. Created with BioRender.com

Interestingly, regarding the role of EBV in the development of MS, particular attention has been paid to treatment strategies in this field. For the first time in 2014, *Pender *et al. applied the vitro-expanded autologous EBV-specific CD8 ( +) T cells directed against viral latent proteins to treat a patient with secondary progressive MS. Their results were promising with no adverse effects and evidence of clinical and MRI improvement. Since then, many efforts have been made in this regard. More ever, there is a possibility that currently available B cell depleting therapies might be regarded as anti-EBV therapies, which deplete circulating memory B cells, the primary site of latent EBV infection [58]. The mechanism of Epstein-Barr Virus-induced CNS damage is given in Fig. 2.

### HTLV-1

The human T-lymphotropic virus type 1 (HTLV1) is a single-stranded RNA virus that belongs to the *Retroviridae* family, the *Orthoretrovirinae* subfamily, and the deltaretrovirus genus, preferentiallyinfects CD4^+^ T cells in vivo [59]. The HTLV1 genome contains diversified structural genes such as Pol, Gag, and Env, encoding the proteins of enzyme and viral structure, regulating genes including Tax, Rex, and accessory genes including p12, p21, p30, p13, and HTLV-1 basic leucine zipper factor (HBZ). HTLV-1 possesses different strategies to evade host immune responses. Among viral genes, Tax and HBZ play an essential function in the pathogenesis of HTLV1-induced diseases.

HTLV1 affects approximately 5–10 million persons worldwide. Most infected individuals remain asymptomatic; however, a portion of HTLV-1-positive individuals will develop HTLV-1-associated myelopathy/tropical spastic paraparesis (HAM/TSP), adult T-cell leukemia/lymphoma (ATLL) disease, and HTLV-1-Associated Uveitis [60]. HAM / TSP is a progressive chronic inflammatory disease of the CNS, which mainly manifests with slowly progressive spastic paraparesis and significant sphincter impairment. Given the similarities with some forms of MS, HAM / TSP might be misdiagnosed with MS. Interestingly, there are few reports on the co-occurrence of MS and HTLV1 [61, 62].

In HAM/TSP patients, the Tax mRNA level is significantly higher than in healthy people [63]. The Tax protein directs ATF/CREB pathway and expresses viral genes [64]. Moreover, Tax through NF-κB activation induces cellular gene transcription and alters HTLV-1-infected cells [64]. It also stimulates T cell activation and proliferation in HAM / TSP patients by increasing the expression of IL-2 genes, IL-2 receptor domain an (IL-2Ra), IL-15, and IL-15Ra [65–69]. Tax-induced elevations in IL-2, IL-9, and IL-15 activate the Jak3/STAT5 axis, and Jak3 blockade has been found to reduce the immunological stimulation in PBMCs in HAM/TSP patients [70]. HBZ inhibits the NF-B pathway and decreases Tax activity via interacting with the CREB/ATF pathway [71]. Numerous studies have shown that in HAM / TSP patients, the activity of Treg cells and the expression of Foxp3 are significantly reduced, which is assumed to be the result of Tax overexpression [72–75]. In addition, recent studies have demonstrated that the Tax protein could inhibit the TGF-β gene expression through disruption of TGF-β signaling expression [76]. Decreased numbers of CD4 + CD25 + Foxp3 + Treg cells were seen in transgenic rodents expressing HTLV-1 Tax that expands an inflammatory arthropathy [77]. It has been revealed that the CD4^+^CCR4^+^ T cells that coexpressed the Th1 marker CXCR3 and produced T-bet and IFN-γ were present in the CSF and spinal cord lesions of HAM/TSP patients [78]. Being activated by IFN-γ, astrocytes release CXCL10, attracting additional CXCR3 + T-cells to the CNS. This situation creates an inflammatory positive feedback loop, accompanied by subsequent fabric damage [79].

### HSV-1

Herpes simplex virus type 1 (HSV-1) is a part of the *herpes simplex* family with a double-stranded DNA-encapsulated virus [80]. HSV-1 is a ubiquitous virus affecting more than 60% of people worldwide. However, only 20 to 40% of infected people show clinical symptoms varying from mild skin involvement to severe peripheral and central nervous system infection [81]. The HSV-1 has two infectious phases, which include the lytic and latent phases. In the latent phase, the infectious virus is produced, while the viral components are not detectable in the individual [82]. During the HSV-1 lytic phase, it expresses an orchestrated of viral genes in the virus-infected cells, including three main categories of gene expression: immediate early gene, early gene, and late gene [83]. On the other hand, HSV-1 encodes several factors to escape the immune system, which causes the virus to persist for a long time. Notably, while during latency there is limited gene expression and no production of viral particles, the viral genome still has potential for reactivation, leading to the production of infectious virions upon the appropriate stimulus [84].

The mechanisms leading to latency and reactivation and which are the viral and host factors controlling these processes are not completely understood. The Us6 gene encodes glycoprotein D, which is a part of HSV construction is required for penetrating the host cells, and inhibiting the apoptosis by activating NF-κB and enhancing NF-κB-dependent anti-apoptotic genes such as FLIP and c-IAP2 [85, 86]. Glycoprotein E has been shown to act as an inhibitor of apoptosis in epithelial cells and is produced by activating ERK1/2, which is associated with the degradation of the Bim protein [87]. ICP22 is another HSV-1 protein that plays a negative role in apoptosis [88]; the deleted ICP22 recombinant HSV-1 induces more apoptosis compere to unmanipolated virus [89]. Another mechanism is disruption of the autophagy process, a process that removes the damaged organs and prevents the accumulation of misfolded proteins, leading to the cellular hemostasis [90]. Animal studies have shown that Atg5 and Atg7 are essential proteins for autophagy; therefore, mice with defects in the Atg5 [91] and Atg7 [92] proteins have provided evidence of neurodegenerative diseases In this regard, HSV1 inhibits the cellular protein synthesis, impeding the virus replication through eukaryotic initiation factor 2a (eIF2a) phosphorylation, which is known to be involved in controlling HSV-1 in neurons [93].

In terms of neurotoxicity, herpes simplex encephalitis (HSE) is considered as the most devastating manifestation of HSV-1. It can occur either in primary infection or upon reactivation from latency. Cytolytic HSV-1 proliferation and immune factors are involved in the development of HSE [94–97]. The intrinsic and innate immune responses are key to protect against HSV-1 infection of the CNS and subsequent pathologies, including HSE. Toll like Receptors (TLRs) are parts of the innate immune system that provide the first line of defense against viral infection. HSV-1 infection in astrocytes activates TLR2 and TLR4, which causes IFN-I expression and an increase in pro-inflammatory cytokines such as IL-6 [98]. TLR3 also plays an important role in controlling HSV-1 infection. It detects viral double-stranded RNA which is produced during HSV-1 replication, inducing the production of type I IFNs [99]. HSV-1 neuro-infection induces the expression of cytokines and pro-inflammatory molecules such as TNF-a, IL-6, IL-8, CCL5, CXCL10, and macrophage inflammatory protein 1a (MIP-1a) in the brain [100, 101]. During the acute phase of HSE, macrophage and neutrophile cells enter the brain, triggering an immune response to eliminate the infection [102, 103]. Penetrated macrophages secrete TNF-α, and microglial cells express IL-1B [104]. Moreover, infiltrating CTL cells detect the HSV-1 glycoprotein B and promote the neural infected cells' death [105]. T lymphocytes are the primary leukocytes in the brain 14 days after infection, and CD8 + T cells express IFN-γ, which cooperates with TNF-α and increases NO-induced neurodegeneration and demyelination [106]. Ideally, the immune response controls the virus. Otherwise, an uncontrolled and excessive immune response might be detrimental.

Interestingly, there is evidence suggesting the potential link between HSV and Alzheimer's disease (AD). HSV-1 DNA has been shown to be co-located with amyloid B in the brain tissue samples of patients with AD. The association between HSV-1 and AD is stronger in individuals carrying the APOE4 allele, one of the strongest genetic risk factors for AD [107]. Moreover, HSV-1 causes the accumulation of AB1-40 and AB1-42 and reduces ABPP levels, hallmarks of AD pathology, which indicates a predisposing factor in AD [108]. There is also evidence of mitochondrial pathway disruption in virus neurotoxicity. Several studies have shown that HSV-1 infection increases the Reactive Oxygen Species (ROS) levels, which have been demonstrated to play a role in development of AD [61, 109]. TLR2.

Regarding the association of HSV1 and MS, the scope of article is inflammatory disorders.

### Influenza

Influenza virus is an enveloped, negative-sense single-stranded RNA virus that consists of 8 parts and is classified as the *orthomyxoviridae* family membership [62]. Influenza virus known as three different subtypes, including A‌, ‌B, and C, whereas influenza A and B are the primary pathogens in humans [110]. Currently, 18 hemagglutinin (HA) subtypes and 11 neuraminidase (NA) subtypes were investigated [111]. HA binds the virus to the sialic acid receptor, which causes the membrane to fuse and enter the cell. NA is a receptor-degrading enzyme that is required for virus release and virus spread [112]. Infections alone cannot induce autoimmune diseases and other factors such as genetics, hormones, and immunity are also involved [113]. However, there is evidence that the infections play a role to induce autoimmune diseases such as Guillain-Barré syndrome, Multiple Sclerosis, and Autism [94]. Influenza has been identified as a trigger for MS. One study found a positive association between the occurrence of influenza and MS [95]. A case–control study also showed that IgG against several viruses, including Influenza A, was higher in MS patients than in controls [96]. However, another study has shown that the Influenza vaccine does not affect the risk of developing MS [97]. Among the reported autoimmune complications, GBS is the most commonly reported autoimmune disease caused by the Influenza vaccine [114]. Sivadan Tardy et al. in France demonstrated that there was a positive correlation between the monthly prevalence of GBS for unknown reasons and the number of Influenza patients. Although influenza serology has low accuracy in diagnosing influenza, there has been a significant association between GBS patients and influenza A and B [115]. In another study in the UK, Tam et al. examined the risk of developing GBS after catching influenza and found that the risk of developing GBS increased within two months of catching the Influenza [116].

The influenza virus genome is detected by TLR7, while during virus replication, double-stranded RNA is detected by TLR3. Activation of the corresponding TLRs by ssRNA or dsRNA activates the signal cascade. TLRs are not required to activate T cells against influenza. However, they induce B cell responses directly and indirectly by INF-α [117], which stimulate B cells to proliferate, switch to the IgG antibody class, and produce autoantibodies. Influenza-infected cells also produce ‌IFN-α, which causes DC to mature and activate T cells. One possible response to autoimmune diseases following influenza infection is to reduce the down-regulation of DC cells, which increases the number of activated cells [118]. Influenza virus can also stimulate pro-inflammatory cytokines such as IL-8, which can cause autoimmune diseases [119]. Another response to autoimmune diseases is "molecular mimicry," which is antigen-dependent, and the immune system responds to similar antigens to microbial segments [120]. GBS is a peripheral nervous system (PNS) autoimmune disease, usually established following infection. GBS, Fisher syndrome (FS), and Bickerstaff brainstem encephalitis (BBE) are considered as GBS-related disease (GBSRD). Anti-glycolipid antibodies are raised in GBSRD and involved in the pathogenesis. The anti-GM1 antibody is found in GBS after infection with C.Jejuni, and the antibody against galactoserbroside is found in neurological diseases after infection with M.Pneumonia [121, 122]. The carbohydrate component of neurons is similar to carbohydrates produced by infectious agents, recommending molecular mimicry is responsible for GBSRD [123, 124]. Although anti-glycolipid antibodies are more abundant in C.Jejuni-induced GBS (GBSRD-C) than influenza-induced GBS and anti-GM1 antibodies are more abundant in Influenza-induced GBSs, anti-GQ1b is significantly higher in influenza-induced GBSRDs [125]. Moreover, Anti-GT1a is also moderately more common in Influenza -induced GBSRD patients [125]. Nachamkin et al. [126] studied the role of the A / NJ / 1976 influenza vaccine in causing an inappropriate immune response in mice. All vaccines against mice were able to induce the production of antibodies against HA, especially GM1. Studies showed that vaccine A / NJ / 1976 and subsequent vaccines had a Glycan layer that antibodies could immunohistochemically stain against GM1. Moreover, viral HA, which usually binds to sialic acid, can form the sialic acid-HA complex, which is destroyed by NA. Low NA levels because incomplete removal of sialic acid from viral HA can eventually mimic the structure of GM1 [126, 127].

### SARS-CoV2

SARS-CoV2 is an enveloped, positive-sense, single-stranded RNA (ssRNA) of the *coronaviridae* family [128–130]. Coronaviruses are classified into four genera: Alphacoronavirus (αCoV), Beta coronavirus (βCoV), Gamma coronavirus (ϒCoV), and Delta coronavirus (δCoV). Human coronaviruses (HCoV) belong to α- and βCoVs [131, 132] and a newly emerged HCoV, SARS-CoV2, was clustered with lineage βCoV [133, 134]. Studies in Covid-19 patients have shown that after the outbreak of SARS-CoV-2, reports of neurological complications such as GBS [135], AD [136], Parkinson disease(PD) [136], and MS have increased [103]. So far, the neurologic manifestations related to SARS-CoV-2 infection were reported widely varied. The pandemic results from COVID-19 revealed that the association between the incidence of Guillain–Barré syndrome (GBS) and previous SARS-CoV-2 infection is not very clear [137]. It was reported that GBS numerate as one of the frequently manifested PNS complications for COVID-19 [138]. The first case of GBS has been reported in a 61-year-old COVID-19-positive woman [131]. Recently, numerous case reports have been reported around the world in COVID-19 patients [139, 140]. Possible association between SARS-CoV-2 vaccination and GBS have been reported in several researches [98, 141]. In various epidemiological studies the association between GBS and SARS-CoV-2 infection have been investigated, as some of these studies finds no association between COVID-19 and GBS [142]. However, in some studies contradictory data have been reported. Palaiodimou L and et al. reported that among 136,746 COVID-19 patients the pooled GBSs prevalence was estimated 15 cases per 100,000. Also, they found that COVID-19 patients had increased odds for demyelinating GBSs subtypes [143]. In one retrospectively study, Restrepo-Vera JL and et al. investigated the relationship between GBS and SARS-CoV-2 infection, as findings showed a clear increase in GBS cases at the expense of a significant number of GBS-S. It was reported that this contradictory findings may be explained by a decrease in the number of cases of GBS associated with other infections due to the wearing mask, hand hygiene, and social distancing [99, 144, 145]. In a retrospective cohort study, Wang L and et al. investigated whether SARS-CoV-2 viral infection is associated with increased risk for AD. Of the 6,245,282 older adults (age ≥ 65 years) enrolled in the study, they found that people with COVID-19 were at significantly increased risk for new diagnosis of AD within 360 days after the initial COVID-19 diagnosis, especially in people age ≥ 85 years and in women [146]. Li S and et al. investigated ecological time-series analysis of AD and PD mortality during the COVID-19 pandemic in the USA. Findings revealed that from March 2020 to March 2022, the number of 41,115 and 10,328 excess deaths have been reported for AD and PD, respectively. This excess mortalities for AD and PD were about 23 and 9 times higher than those aged 55–84 years, respectively. Also, it was reported that females had a three-time higher excess mortality of AD than males [100]. In a retrospective cross-sectional study, Gilstrap L and et al. investigated the association between mortality among older adults with Alzheimer disease and related dementias (ADRD). Findings revealed that compared with 2019, adjusted mortality in 2020 was 12.4% higher among enrollees without ADRD and 25.7% higher among all enrollees with ADRD among 53 640 888 Medicare with 65 years of age or older [101]. Related to the MS, we did not find any retrospective cohort study with large size to validate the association between the increase of the MS at the age of COVID-19 disease. Most of the studies focused on the prevalence of COVID-19 infection in patients with multiple sclerosis (MS), and did not clearly investigate the increase of the MS at the age of COVID-19 disease. Some data support that the hospitalization rate is higher among MS patients based on COVID-19 infection [102]. It was reported that CNS demyelination has occurred shortly after COVID-19, suggesting that these symptoms could be the result of neurological damage following SARS-CoV-2 infection, or they could be coincidental, from causes such as secondary systemic complications or side effects of drug treatment [102, 103].

SARS-CoV-2 requires the angiotensin-converting enzyme 2 (ACE2) as a functional receptor to penetrate cells. The virus binds to the ACE2 receptor via the spike, although the virus has also been observed to use Basigin (CD147) and Neuropilin-1 (NRP1) as receptors [104]. After SARS-Cov-2 infecting the cells, PRRs identified PAMP and DAMP of viruses and induced inflammatory reaction [18]. Following SARS-COv-2 entrance, infected neural cell can kill directly or indirectly though using immune system. Moreover, neurodegenerative process was conducted as an acute and chronic phase. Indirect damage proceeded in several mechanisms, including molecular mimicry, cytokine storm which induce self-antigens productions, and autoantibodies production.

**Fig. 3:**
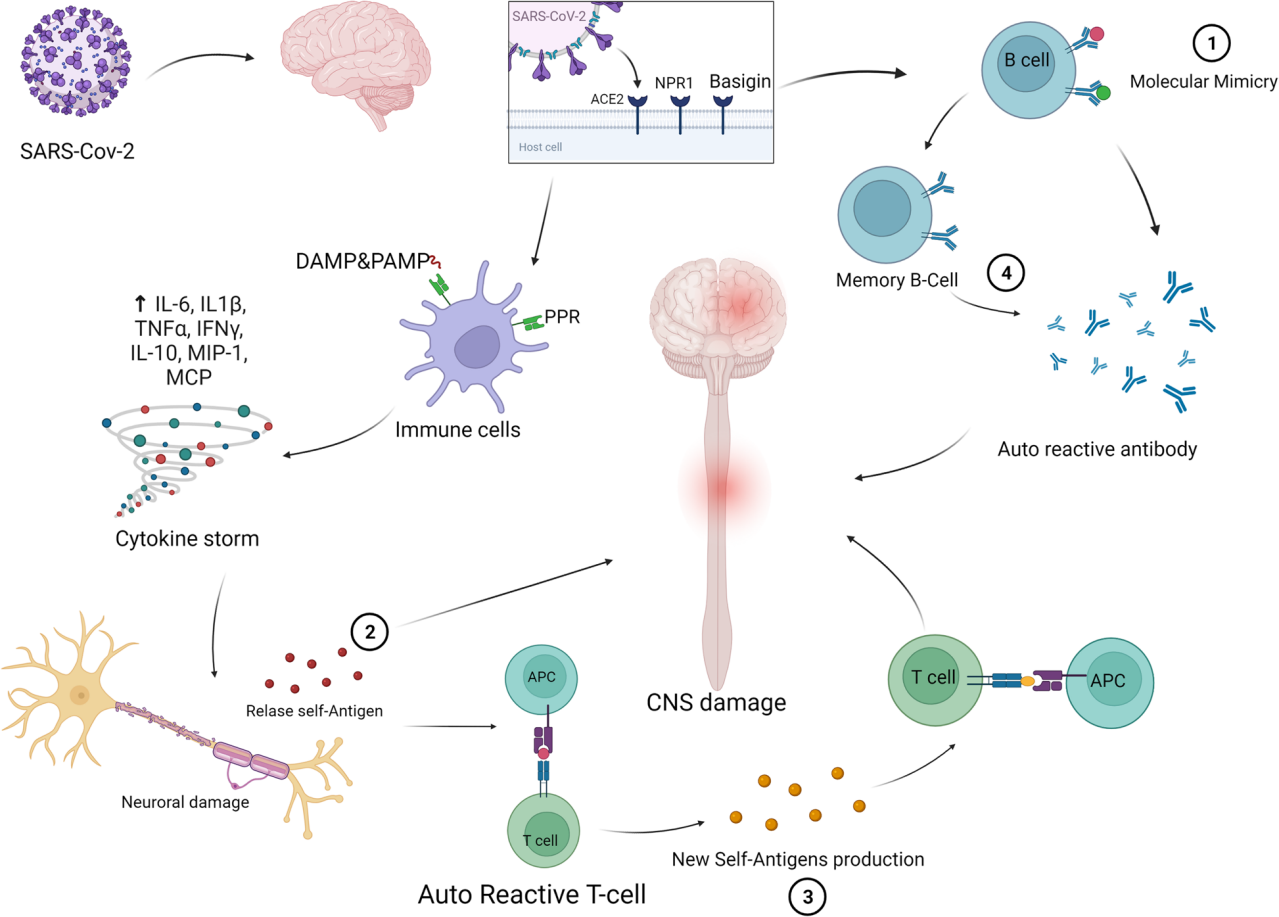
The mechanism SARS-Cov-2 induces neurological autoimmune system. SARS-Cov-2 enters the nervous system through different receptors. The viruses induce autoimmune reactions through several pathways. 1) The B- cells produce autoreactive antibodies against SARS-Cov-2, which damaged the normal tissues. 2) The recognized virus through PRR induces cytokine storm, resulting in damage to the healthy tissue and new antigens that, releases activate autoreactive T-cells. 3) Subsequently, the T-cell reaction destroys tissues and produce new self-antigens, which induce more autoreactive T-cells reaction. 4) The memory B-cells produce long-term autoantibodies in the absence of virus, which was associated with sustained CNS damage—created with BioRender.com

Molecular mimicry is the one of the main proposed mechanism involved in neurodegeneration which can cause the immune system to become overactive in autoimmune diseases are similar to those of the immune response against SARS-CoV2 [105]. Molecular mimicry involves the structural similarity of SARS-CoV2 antigens to their self-antigens, which activate the B cells and T cells against human-like proteins, which causes autoimmune diseases [105]. Molecular mimicry is the most common cause considered for GBS. Anti-ganglioside antibodies were identified in the 50 to 85 percent of GBS patients. The rise in anti-ganglioside antibodies in GBS patients with Covid-19 remains unclear; however, Dufour, et al. found the presence of anti-GM1 antibodies in GBS patients infected with Covid-19 [106]. Furthermore, among 58 Covid-19 patients with neurological symptoms, SARS-CoV-2 was identified in the CSF of only two patients [147] the inability to identify SARS-CoV-2 genome in the CSF of most patients indicates that direct virus attack does not involved in the autoimmunity [104]. Another mechanism for autoimmune diseases in Covid-19 patients is the aberrant immune system's response to SARS-CoV2, which triggers the inflammatory cytokines and chemokines production, including IL-1B, IL-6, IL-8, TNF-a, IFN-γ, Granulocyte colony-stimulating factor (G-CSF), induced protein 10 (IP-10), monocyte chemoattractant protein 1 (MCP-1), and macrophage inflammatory protein 1a (MIP-1a) [18]. This aberrant immune reaction to the virus triggers the cytokine storm and production of inflammatory conditions which induce disruption of self-tissue and produced SARS-Cov-2 antigens-mimicking which is involved in the pathogenesis neurodegenerative disorders [148]. The pathogenesis of MS is not fully understood; however, several studies demonstrated that immune response and inflammation lead to MS. Studies have shown that inflammatory cytokines such as IL-12, IL-17, IFN-γ, and TNF-a are significantly higher in the CSF of MS patients [149]. In Covid-19 patients, Th17 levels increase, which regulates inflammatory conditions by increasing IL-6 and IL-23, indicating the pivotal role of this cell in the production of Cytokines Storm, which provides the requirements for MS [150]. Following the production of these self-antigens due to the cytokine storm, antigen presenting cells promote the T cells and induced autoreactive responses which results in further undertaken neurodegeneration. Moreover, upon self-tissue damaged, de novo new self-epitopes are produced which further induce autoreactive T cell activation and sustained neurodegenerative process. Additionally, memory process of immune system encourages production of further antibodies versus different nervous system tissue, such as blood–brain barrier and myelin sheet which results in prolonged and severe neurodegenerative process in the overed-Covid-19 state [151] (Fig. 3).

Following the emergence of covid-19, vaccination against SARS-Cov-2 is considered as an effective strategy for preventing infected to covid-19 and decreasing the severity and mortality of patients infected by SARS-Cov-2. However, some clinical studies reported the patients with novel neurological autoimmune diseases who is diagnosed after receiving covid-19 vaccines. Rinaldi et al. reported the patients with CNS inflammatory demyelinating events following covid-19 vaccine administration. They showed that covid-19 vaccine can induce acute transverse myelitis, MS, acute demyelinating encephalomyelitis (ADEM), and neuromyelitis optica spectrum disorder (NMOSD) [152]. Moreover, Abdelhady et al. reported 65 patients who developed encephalitis following covid-19 vaccines [153]. Despite first presentation of MS after covid-19 vaccine was reported in some studies [154–157], Stefanou et al. showed that Covid-19 vaccination is not associated with increasing the risk of relapse of patients with MS irrespective to the type of covid-19 vaccines [158], and therefore, most of the patients with MS are willing to receive covie-19 vaccine [159].

## Conclusion

Autoimmune diseases are frequently developed based on the interaction between several factors, including genetic susceptibility, aberrant immune response, and environmental factors such as infections. The viral infection seems more critical to inducing autoimmune disease disorders. The exact level of involvement of these factors was not elucidated [160]; however, several studies revealed that viruses could caus [161] or exacerbate [162, 163] autoimmune disease. In contrast, accumulating data suggest that viruses regulate the immune response and protect against the onset of autoimmune diseases [3]. Viruses can induce several immune pathways, resulting in an aberrant immune response. The mechanisms, including bystander activation, cryptic antigens presentation, epitope spreading, and molecular mimicry, were considered the main pathways to induce autoimmune reactions in general autoimmune diseases [160]. Based on the complex nature of the nervous system, it is expected that viruses cause autoimmune diseases of the nervous system in more complex ways. In this review, we summarized the investigated mechanisms of viruses-induced neurological autoimmunity. We showed that viruses promote different gene expressions and cause immune system over-activation and cytokine storms alongside previously known mechanisms of general autoimmune disease induced by viruses, resulting in immune-mediated tissue injury. Conclusively interactions of Host (Genetic and Immune system) and viral factors can determine how the immune system induces effective or pathologic response.

## Data Availability

The datasets used in the current study are available from the corresponding author on reasonable request.

## References

[1] Fairweather D, Root-Bernstein R. Autoimmune Disease: Mechanisms. 2007.

[2] Arleevskaya MI, Manukyan G, Inoue R, Aminov R (2017). Editorial: microbial and environmental factors in autoimmune and inflammatory diseases. Front Immunol.

[3] Lerner A, Arleevskaya M, Schmiedl A, Matthias T (2017). Microbes and viruses are bugging the gut in celiac disease. Are they friends or foes?. Front Microbiol.

[4] Klase ZA, Khakhina S, Schneider Ade B, Callahan MV, Glasspool-Malone J, Malone R (2016). Zika Fetal Neuropathogenesis: etiology of a viral syndrome. PLoS Negl Trop Dis.

[5] Eaton WW, Rose NR, Kalaydjian A, Pedersen MG, Mortensen PB (2007). Epidemiology of autoimmune diseases in Denmark. J Autoimmun.

[6] Theofilopoulos AN, Kono DH, Baccala R (2017). The multiple pathways to autoimmunity. Nat Immunol.

[7] McKeon A, Pittock SJ (2011). Paraneoplastic encephalomyelopathies: pathology and mechanisms. Acta Neuropathol.

[8] Bhagavati S (2021). Autoimmune disorders of the nervous system: pathophysiology, clinical features, and therapy. Front Neurol.

[9] Croxford JL, Olson JK, Miller SD (2002). Epitope spreading and molecular mimicry as triggers of autoimmunity in the Theiler's virus-induced demyelinating disease model of multiple sclerosis. Autoimmun Rev.

[10] Fujinami RS, Herrath MGv, Christen U, Whitton JL (2006). Molecular mimicry, bystander activation, or viral persistence: infections and autoimmune disease. Clin Microbiol Rev.

[11] Getts DR, Chastain EM, Terry RL, Miller SD (2013). Virus infection, antiviral immunity, and autoimmunity. Immunol Rev.

[12] Polepole P, Bartenslager A, Liu Y, Petro TM, Fernando S, Zhang L (2021). Epstein-Barr virus-immortalized B lymphocytes exacerbate experimental autoimmune encephalomyelitis in xenograft mice. J Med Virol.

[13] Traggiai E (2012). Immortalization of human B cells: analysis of B cell repertoire and production of human monoclonal antibodies. Methods Mol Biol.

[14] Plosa EJ, Esbenshade JC, Fuller MP, Weitkamp JH (2012). Cytomegalovirus infection. Pediatr Rev.

[15] Griffiths P, Baraniak I, Reeves M (2015). The pathogenesis of human cytomegalovirus. J Pathol.

[16] Gugliesi F, Pasquero S, Griffante G, Scutera S, Albano C, Pacheco SFC (2021). Human Cytomegalovirus and Autoimmune Diseases: Where are we?. Viruses.

[17] Alari-Pahissa E, Moreira A, Zabalza A, Alvarez-Lafuente R, Munteis E, Vera A (2018). Low cytomegalovirus seroprevalence in early multiple sclerosis: a case for the ‘hygiene hypothesis’?. Eur J Neurol.

[18] Ragab D, et al. The COVID-19 Cytokine Storm; What We Know So Far. Front Immunol. 2020;11:1446. 10.3389/fimmu.2020.01446 PMC7308649 32612617

[19] Zheng MM, Zhang XH (2014). Cross-reactivity between human cytomegalovirus peptide 981–1003 and myelin oligodendroglia glycoprotein peptide 35–55 in experimental autoimmune encephalomyelitis in Lewis rats. Biochem Biophys Res Commun.

[20] Milovanovic J, Popovic B, Milovanovic M, Kvestak D, Arsenijevic A, Stojanovic B (2017). Murine cytomegalovirus infection induces susceptibility to EAE in resistant BALB/c Mice. Front Immunol.

[21] t Hart BA, Jagessar SA, Haanstra K, Verschoor E, Laman JD, Kap YS (2013). The primate EAE model points at EBV-infected B Cells as a preferential therapy target in multiple sclerosis. Front Immunol.

[22] Vanheusden M, Broux B, Welten SPM, Peeters LM, Panagioti E, Van Wijmeersch B (2017). Cytomegalovirus infection exacerbates autoimmune mediated neuroinflammation. Sci Rep.

[23] Petersen LR, Jamieson DJ, Powers AM, Honein MA (2016). Zika Virus. N Engl J Med.

[24] Shi Y, Gao GF (2017). Structural Biology of the Zika Virus. Trends Biochem Sci.

[25] Filgueiras IS, Torrentes de Carvalho A, Cunha DP, Mathias da Fonseca DL, El Khawanky N, Freire PP (2021). The clinical spectrum and immunopathological mechanisms underlying ZIKV-induced neurological manifestations. PLoS Negl Trop Dis.

[26] Alves-Leon SV, Lima MDR, Nunes PCG, Chimelli LMC, Rabelo K, Nogueira RMR (2019). Zika virus found in brain tissue of a multiple sclerosis patient undergoing an acute disseminated encephalomyelitis-like episode. Mult Scler.

[27] Alves-Leon SV, Fontes-Dantas FL, Rueda-Lopes FC. Chapter 18 - Neurological manifestations similar to multiple sclerosis in adults after Zika virus infection, in Zika Virus Biology, Transmission, and Pathology, Martin CR, et al., editors. Academic Press. 2021. p. 199–207.

[28] Araujo AQ, Silva MT, Araujo AP (2016). Zika virus-associated neurological disorders: a review. Brain.

[29] Schultz V, et al. Zika Virus Infection Leads to Demyelination and Axonal Injury in Mature CNS Cultures. Viruses. 2021;13(1). 10.3390/v13010091 PMC7827345 33440758

[30] Tricarico PM, Caracciolo I, Crovella S, D'Agaro P (2017). Zika virus induces inflammasome activation in the glial cell line U87-MG. Biochem Biophys Res Commun.

[31] Ojha CR, Rodriguez M, Karuppan MKM, Lapierre J, Kashanchi F, El-Hage N (2019). Toll-like receptor 3 regulates Zika virus infection and associated host inflammatory response in primary human astrocytes. PLoS ONE.

[32] Acosta-Ampudia Y, Monsalve DM, Castillo-Medina LF, Rodríguez Y, Pacheco Y, Halstead S (2018). Autoimmune neurological conditions associated with Zika virus infection. Front Mol Neurosci.

[33] Clé M, Desmetz C, Barthelemy J, Martin M-F, Constant O, Maarifi G (2020). Zika virus infection promotes local inflammation, cell adhesion molecule upregulation, and leukocyte recruitment at the blood-brain barrier. mbio.

[34] Papa MP, Meuren LM, Coelho SVA, Lucas CGO, Mustafá YM, Lemos Matassoli F (2017). Zika virus infects, activates, and crosses brain microvascular endothelial cells, without barrier disruption. Front Microbiol.

[35] Laing KJ, Ouwendijk WJD, Koelle DM, Verjans G (2018). Immunobiology of Varicella-Zoster Virus Infection. J Infect Dis.

[36] Rice EM, Thakolwiboon S, Avila M (2021). Geographic heterogeneity in the association of varicella-zoster virus seropositivity and multiple sclerosis: a systematic review and meta-analysis. Mult Scler Relat Disord.

[37] Kang JH, Sheu JJ, Lin HC (2010). Increased risk of Guillain-Barré Syndrome following recent herpes zoster: a population-based study across Taiwan. Clin Infect Dis.

[38] Najafi S, Ghane M, Yousefzadeh-Chabok S, Amiri M (2016). The High Prevalence of the Varicella Zoster Virus in Patients With Relapsing-Remitting Multiple Sclerosis: A Case-Control Study in the North of Iran. Jundishapur J Microbiol.

[39] Karampoor S, Zahednasab H, Ramagopalan S, Mehrpour M, Etemadifar M, Alsahebfosoul F (2017). Cytomegalovirus and varicella zoster virus seropositivity of Iranian patients with multiple sclerosis: a population-based study. J Neuroimmunol.

[40] Esmaili K, Amini K (2018). Detection of human herpes virus type 6 and varicella zoster virus in the urine of patients with multiple sclerosis in Kerman Province Iran. Neurosci J Shefaye Khatam.

[41] Mancuso R, Delbue S, Borghi E, Pagani E, Calvo MG, Caputo D (2007). Increased prevalence of varicella zoster virus DNA in cerebrospinal fluid from patients with multiple sclerosis. J Med Virol.

[42] Sotelo J, Martínez-Palomo A, Ordoñez G, Pineda B (2008). Varicella-zoster virus in cerebrospinal fluid at relapses of multiple sclerosis. Ann Neurol.

[43] Kattimani Y, Veerappa AM (2018). Complex interaction between mutant HNRNPA1 and gE of varicella zoster virus in pathogenesis of multiple sclerosis. Autoimmunity.

[44] Ascherio A, Munger KL (2010). Epstein-barr virus infection and multiple sclerosis: a review. J Neuroimmune Pharmacol.

[45] Cohen JI (2000). Epstein-Barr virus infection. N Engl J Med.

[46] Albanese M, Tagawa T, Bouvet M, Maliqi L, Lutter D, Hoser J (2016). Epstein-Barr virus microRNAs reduce immune surveillance by virus-specific CD8<sup>+</sup> T cells. Proc Natl Acad Sci.

[47] Nociti V, Frisullo G, Marti A, Luigetti M, Iorio R, Patanella AK (2010). Epstein-Barr virus antibodies in serum and cerebrospinal fluid from multiple sclerosis, chronic inflammatory demyelinating polyradiculoneuropathy and amyotrophic lateral sclerosis. J Neuroimmunol.

[48] Santiago O, Gutierrez J, Sorlozano A, Dios Luna J, Villegas E, Fernandez O (2010). Relation between Epstein-Barr virus and multiple sclerosis: analytic study of scientific production. Eur J Clin Microbiol Infect Dis.

[49] Al-Temaimi R, Alroughani R, Jacob S, Al-Mulla F (2015). Gender influence in EBV antibody response in multiple sclerosis patients from Kuwait. J Neuroimmunol.

[50] Hassani A, Corboy JR, Al-Salam S, Khan G (2018). Epstein-Barr virus is present in the brain of most cases of multiple sclerosis and may engage more than just B cells. PLoS ONE.

[51] Robinson WH, Steinman L (2022). Epstein-Barr virus and multiple sclerosis. Science.

[52] Mameli G, Cocco E, Frau J, Marrosu MG, Sechi LA (2016). Epstein Barr Virus and Mycobacterium avium subsp. paratuberculosis peptides are recognized in sera and cerebrospinal fluid of MS patients. Sci Rep.

[53] Jog NR, McClain MT, Heinlen LD, Gross T, Towner R, Guthridge JM (2020). Epstein Barr virus nuclear antigen 1 (EBNA-1) peptides recognized by adult multiple sclerosis patient sera induce neurologic symptoms in a murine model. J Autoimmun.

[54] Tengvall K, Huang J, Hellström C, Kammer P, Biström M, Ayoglu B (2019). Molecular mimicry between Anoctamin 2 and Epstein-Barr virus nuclear antigen 1 associates with multiple sclerosis risk. Proc Natl Acad Sci U S A.

[55] Pender MP, Csurhes PA, Burrows JM, Burrows SR (2017). Defective T-cell control of Epstein-Barr virus infection in multiple sclerosis. Clin Transl Immunol.

[56] Guerrera G, Ruggieri S, Picozza M, Piras E, Gargano F, Placido R (2020). EBV-specific CD8 T lymphocytes and B cells during glatiramer acetate therapy in patients with MS. Neurol Neuroimmunol Neuroinflammation.

[57] Shi Y, Lutz CT (2002). Interferon–gamma control of EBV-transformed B cells: a role for CD8+ T cells that poorly kill EBV-infected cells. Viral Immunol.

[58] Pender MP, Csurhes PA, Smith C, Beagley L, Hooper KD, Raj M (2014). Epstein-Barr virus-specific adoptive immunotherapy for progressive multiple sclerosis. Mult Scler (Houndmills, Basingstoke, England).

[59] Cook LB, Rowan AG, Melamed A, Taylor GP, Bangham CRM (2012). HTLV-1–infected T cells contain a single integrated provirus in natural infection. Blood.

[60] Proietti FA, Carneiro-Proietti AB, Catalan-Soares BC, Murphy EL (2005). Global epidemiology of HTLV-I infection and associated diseases. Oncogene.

[61] Schachtele SJ, Hu S, Little MR, Lokensgard JR (2010). Herpes simplex virus induces neural oxidative damage via microglial cell Toll-like receptor-2. J Neuroinflammation.

[62] Hutchinson EC, Fodor E (2013). Transport of the influenza virus genome from nucleus to nucleus. Viruses.

[63] Yamano Y, Nagai M, Brennan M, Mora CA, Soldan SS, Tomaru U (2002). Correlation of human T-cell lymphotropic virus type 1 (HTLV-1) mRNA with proviral DNA load, virus-specific CD8+ T cells, and disease severity in HTLV-1–associated myelopathy (HAM/TSP). Blood.

[64] Li XH, Gaynor RB (1999). Regulation of NF-kappaB by the HTLV-1 Tax protein. Gene Expr.

[65] Siekevitz M, Feinberg MB, Holbrook N, Wong-Staal F, Greene WC (1987). Activation of interleukin 2 and interleukin 2 receptor (Tac) promoter expression by the trans-activator (tat) gene product of human T-cell leukemia virus, type I. Proc Natl Acad Sci U S A.

[66] Cross SL, Feinberg MB, Wolf JB, Holbrook NJ, Wong-Staal F, Leonard WJ (1987). Regulation of the human interleukin-2 receptor alpha chain promoter: activation of a nonfunctional promoter by the transactivator gene of HTLV-I. Cell.

[67] Azimi N, Brown K, Bamford RN, Tagaya Y, Siebenlist U, Waldmann TA (1998). Human T cell lymphotropic virus type I Tax protein trans-activates interleukin 15 gene transcription through an NF-kappaB site. Proc Natl Acad Sci U S A.

[68] Mariner JM, Lantz V, Waldmann TA, Azimi N (2001). Human T cell lymphotropic virus type I Tax activates IL-15R alpha gene expression through an NF-kappa B site. J Immunol.

[69] Waldmann TA (2006). The biology of interleukin-2 and interleukin-15: implications for cancer therapy and vaccine design. Nat Rev Immunol.

[70] Ju W, Zhang M, Jiang JK, Thomas CJ, Oh U, Bryant BR (2011). CP-690,550, a therapeutic agent, inhibits cytokine-mediated Jak3 activation and proliferation of T cells from patients with ATL and HAM/TSP. Blood.

[71] Matsuoka M, Jeang KT (2011). Human T-cell leukemia virus type 1 (HTLV-1) and leukemic transformation: viral infectivity, Tax HBZ and therapy. Oncogene.

[72] Yamano Y, Takenouchi N, Li HC, Tomaru U, Yao K, Grant CW (2005). Virus-induced dysfunction of CD4+CD25+ T cells in patients with HTLV-I-associated neuroimmunological disease. J Clin Invest.

[73] Oh U, Grant C, Griffith C, Fugo K, Takenouchi N, Jacobson S (2006). Reduced Foxp3 protein expression is associated with inflammatory disease during human t lymphotropic virus type 1 Infection. J Infect Dis.

[74] Michaëlsson J, Barbosa HM, Jordan KA, Chapman JM, Brunialti MK, Neto WK, Nukui Y, Sabino EC, Chieia MA, Oliveira AS, Nixon DF (2008). The frequency of CD127low expressing CD4+ CD25high T regulatory cells is inversely correlated with human T lymphotrophic virus type-1 (HTLV-1) proviral load in HTLV-1-infection and HTLV-1-associated myelopathy/tropical spastic paraparesis. BMC Immunol.

[75] Araya N, et al. Human T-lymphotropic virus type 1 (HTLV-1) and regulatory T cells in HTLV-1-associated neuroinflammatory disease. Viruses. 2011;3(9):1532–48. 10.3390/v3091532 PMC3187691 21994794

[76] Grant C, Oh U, Yao K, Yamano Y, Jacobson S (2008). Dysregulation of TGF-beta signaling and regulatory and effector T-cell function in virus-induced neuroinflammatory disease. Blood.

[77] Araya N, Sato T, Yagishita N, Ando H, Utsunomiya A, Jacobson S (2011). Human T-lymphotropic virus type 1 (HTLV-1) and regulatory T cells in HTLV-1-associated neuroinflammatory disease. Viruses.

[78] Araya N, Sato T, Ando H, Tomaru U, Yoshida M, Coler-Reilly A (2014). HTLV-1 induces a Th1-like state in CD4+CCR4+ T cells. J Clin Invest.

[79] Yamano Y, Coler-Reilly A (2017). HTLV-1 induces a Th1-like state in CD4<sup>+</sup><span style="display:inline-block;width:0.10em;"><!----></span>CCR4<sup>+</sup> T cells that produces an inflammatory positive feedback loop via astrocytes in HAM/TSP. J Neuroimmunol..

[80] Boehmer P, Nimonkar A (2003). Herpes virus replication. IUBMB Life.

[81] Looker KJ, Magaret AS, May MT, Turner KME, Vickerman P, Gottlieb SL (2015). Global and Regional Estimates of Prevalent and Incident Herpes Simplex Virus Type 1 Infections in 2012. PLoS ONE.

[82] Whitley RJ, Roizman B (2001). Herpes simplex virus infections. The Lancet.

[83] Honess RW, Roizman B (1974). Regulation of herpesvirus macromolecular synthesis. I. Cascade regulation of the synthesis of three groups of viral proteins. J Virol.

[84] Perng G-C, Jones C, Ciacci-Zanella J, Stone M, Henderson G, Yukht A (2000). Virus-induced neuronal apoptosis blocked by the herpes simplex virus latency-associated transcript. Science.

[85] Medici MA, Sciortino MT, Perri D, Amici C, Avitabile E, Ciotti M (2003). Protection by Herpes Simplex Virus Glycoprotein D against Fas-mediated Apoptosis: ROLE OF NUCLEAR FACTOR & #x3ba;B *. J Biol Chem.

[86] Marino-Merlo F, Papaianni E, Medici MA, Macchi B, Grelli S, Mosca C (2016). HSV-1-induced activation of NF-κB protects U937 monocytic cells against both virus replication and apoptosis. Cell Death Dis.

[87] Pontes MS, Van Waesberghe C, Nauwynck H, Verhasselt B, Favoreel HW (2016). Pseudorabies virus glycoprotein gE triggers ERK1/2 phosphorylation and degradation of the pro-apoptotic protein Bim in epithelial cells. Virus Res.

[88] Nguyen ML, Kraft RM, Blaho JA (2005). African green monkey kidney Vero cells require de novo protein synthesis for efficient herpes simplex virus 1-dependent apoptosis. Virology.

[89] Nguyen ML, Blaho JA (2006). Apoptosis During Herpes Simplex Virus Infection. Adv Virus Res..

[90] Klionsky DJ, Cuervo AM, Dunn JWA, Levine B, van der Klei IJ, Seglen PO (2007). How shall I eat thee?. Autophagy.

[91] Hara T, Nakamura K, Matsui M, Yamamoto A, Nakahara Y, Suzuki-Migishima R (2006). Suppression of basal autophagy in neural cells causes neurodegenerative disease in mice. Nature.

[92] Komatsu M, Waguri S, Chiba T, Murata S, Iwata J-i, Tanida I (2006). Loss of autophagy in the central nervous system causes neurodegeneration in mice. Nature.

[93] O'Connell D, Liang C (2016). Autophagy interaction with herpes simplex virus type-1 infection. Autophagy.

[94] Molina V, Shoenfeld Y (2005). Infection, vaccines and other environmental triggers of autoimmunity. Autoimmunity.

[95] Kaźmierski R, Wender M, Guzik P, Zielonka D (2004). Association of influenza incidence with multiple sclerosis onset. Folia Neuropathol.

[96] Krone B, Pohl D, Rostasy K, Kahler E, Brunner E, Oeffner F (2008). Common infectious agents in multiple sclerosis: a case-control study in children. Mult Scler.

[97] DeStefano F, Verstraeten T, Jackson LA, Okoro CA, Benson P, Black SB (2003). Vaccinations and risk of central nervous system demyelinating diseases in adults. Arch Neurol.

[98] Patone M, Handunnetthi L, Saatci D, Pan J, Katikireddi SV, Razvi S (2021). Neurological complications after first dose of COVID-19 vaccines and SARS-CoV-2 infection. Nat Med.

[99] Murillo-Zamora E, Guzmán-Esquivel J, Sánchez-Piña RA, Cedeño-Laurent G, Delgado-Enciso I, Mendoza-Cano O (2020). Physical distancing reduced the incidence of influenza and supports a favorable impact on SARS-CoV-2 spread in Mexico. J Infect Dev Ctries.

[100] Li S, Han L, Shi H, Chong MK, Zhao S, Ran J (2022). Excess deaths from Alzheimer’s disease and Parkinson’s disease during the COVID-19 pandemic in the USA. Age Ageing.

[101] Gilstrap L, Zhou W, Alsan M, Nanda A, Skinner JS (2022). Trends in mortality rates among Medicare enrollees with Alzheimer disease and related dementias before and during the early phase of the COVID-19 pandemic. JAMA Neurol.

[102] Moghadasi AN, Mirmosayyeb O, Barzegar M, Sahraian MA, Ghajarzadeh M (2021). The prevalence of COVID-19 infection in patients with multiple sclerosis (MS): a systematic review and meta-analysis. Neurol Sci.

[103] Pacheco-Herrero M, Soto-Rojas LO, Harrington CR, Flores-Martinez YM, Villegas-Rojas MM, León-Aguilar AM (2021). Elucidating the neuropathologic mechanisms of SARS-CoV-2 infection. Front Neurol.

[104] Iadecola C, Anrather J, Kamel H (2020). Effects of COVID-19 on the nervous system. Cell.

[105] Marino Gammazza A, Légaré S, Lo Bosco G, Fucarino A, Angileri F, Oliveri M (2021). Molecular mimicry in the post-COVID-19 signs and symptoms of neurovegetative disorders?. Lancet Microbe.

[106] Dufour C, Co T-K, Liu A (2021). GM1 ganglioside antibody and COVID-19 related Guillain Barre syndrome – a case report, systemic review and implication for vaccine development. Brain Behav Immun Health.

[107] Wozniak M, Mee A, Itzhaki R (2009). Herpes simplex virus type 1 DNA is located within Alzheimer's disease amyloid plaques. J Pathol.

[108] Wozniak MA, Itzhaki RF, Shipley SJ, Dobson CB (2007). Herpes simplex virus infection causes cellular β-amyloid accumulation and secretase upregulation. Neurosci Lett.

[109] Kavouras JH, Prandovszky E, Valyi-Nagy K, Kovacs SK, Tiwari V, Kovacs M (2007). Herpes simplex virus type 1 infection induces oxidative stress and the release of bioactive lipid peroxidation by-products in mouse P19N neural cell cultures. J Neurovirol.

[110] Yoon SW, Webby RJ, Webster RG (2014). Evolution and ecology of influenza A viruses. Curr Top Microbiol Immunol.

[111] Kosik I, Yewdell JW (2019). Influenza hemagglutinin and neuraminidase: Yin-Yang proteins coevolving to Thwart immunity. Viruses.

[112] Pica N, Palese P (2013). Toward a universal influenza virus vaccine: prospects and challenges. Annu Rev Med.

[113] Toplak N, Avcin T (2009). Influenza and autoimmunity. Ann N Y Acad Sci.

[114] Haber P, DeStefano F, Angulo FJ, Iskander J, Shadomy SV, Weintraub E (2004). Guillain-Barré syndrome following influenza vaccination. JAMA.

[115] Sivadon-Tardy V, Orlikowski D, Porcher R, Sharshar T, Durand M-C, Enouf V (2009). Guillain-Barré syndrome and influenza virus infection. Clin Infect Dis.

[116] Tam CC, O'Brien SJ, Petersen I, Islam A, Hayward A, Rodrigues LC (2007). Guillain-Barré syndrome and preceding infection with campylobacter, influenza and Epstein-Barr virus in the general practice research database. PLoS ONE.

[117] Heer AK, Shamshiev A, Donda A, Uematsu S, Akira S, Kopf M (2007). TLR signaling fine-tunes anti-influenza B cell responses without regulating effector T cell responses. J Immunol.

[118] Marks DJ, Mitchison NA, Segal AW, Sieper J (2006). Can unresolved infection precipitate autoimmune disease?. Curr Top Microbiol Immunol.

[119] Brown AS, Begg MD, Gravenstein S, Schaefer CA, Wyatt RJ, Bresnahan M (2004). Serologic evidence of prenatal influenza in the etiology of schizophrenia. Arch Gen Psychiatry.

[120] Fiore AE, Shay DK, Broder K, Iskander JK, Uyeki TM, Mootrey G (2008). Prevention and control of influenza: recommendations of the Advisory Committee on Immunization Practices (ACIP), 2008. MMWR Recomm Rep.

[121] Hughes RA, Hadden RD, Gregson NA, Smith KJ (1999). Pathogenesis of Guillain-Barré syndrome. J Neuroimmunol.

[122] Kuwahara M, Samukawa M, Ikeda T, Morikawa M, Ueno R, Hamada Y (2017). Characterization of the neurological diseases associated with Mycoplasma pneumoniae infection and anti-glycolipid antibodies. J Neurol.

[123] Ang CW, Laman JD, Willison HJ, Wagner ER, Endtz HP, De Klerk MA (2002). Structure of Campylobacter jejuni lipopolysaccharides determines antiganglioside specificity and clinical features of Guillain-Barré and Miller Fisher patients. Infect Immun.

[124] Kusunoki S, Shiina M, Kanazawa I (2001). Anti-Gal-C antibodies in GBS subsequent to mycoplasma infection: evidence of molecular mimicry. Neurology.

[125] Yamana M, Kuwahara M, Fukumoto Y, Yoshikawa K, Takada K, Kusunoki S (2019). Guillain-Barré syndrome and related diseases after influenza virus infection. Neurol Neuroimmunol Neuroinflammation.

[126] Nachamkin I, Shadomy SV, Moran AP, Cox N, Fitzgerald C, Ung H (2008). Anti-ganglioside antibody induction by swine (A/NJ/1976/H1N1) and other influenza vaccines: insights into vaccine-associated Guillain-Barré syndrome. J Infect Dis.

[127] Kendal AP, Bozeman FM, Ennis FA (1980). Further studies of the neuraminidase content of inactivated influenza vaccines and the neuraminidase antibody responses after vaccination of immunologically primed and unprimed populations. Infect Immun.

[128] He F, Deng Y, Li W (2020). Coronavirus disease 2019: what we know?. J Med Virol.

[129] Nordvig AS, Fong KT, Willey JZ, Thakur KT, Boehme AK, Vargas WS (2021). Potential Neurologic Manifestations of COVID-19. Neurol Clin Pract..

[130] Sahin A-R, Erdogan A, Agaoglu PM, Dineri Y, Cakirci A-Y, Senel M-E (2020). 2019 novel coronavirus (COVID-19) outbreak: a review of the current literature. EJMO.

[131] Zhao H, Shen D, Zhou H, Liu J, Chen S (2020). Guillain-Barré syndrome associated with SARS-CoV-2 infection: causality or coincidence?. Lancet Neurol.

[132] Paules CI, Marston HD, Fauci AS (2020). Coronavirus infections—more than just the common cold. JAMA.

[133] Gerges Harb J, Noureldine HA, Chedid G, Eldine MN, Abdallah DA, Chedid NF (2020). SARS, MERS and COVID-19: clinical manifestations and organ-system complications: a mini review. Pathog Dis.

[134] Wang L, Wang Y, Ye D, Liu Q (2020). Review of the 2019 novel coronavirus (SARS-CoV-2) based on current evidence. Int J Antimicrob Agents.

[135] Sharma K, Tengsupakul S, Sanchez O, Phaltas R, Maertens P (2019). Guillain-Barré syndrome with unilateral peripheral facial and bulbar palsy in a child: a case report. SAGE Open Med Case Rep.

[136] Wang F, Kream RM, Stefano GB (2020). Long-term respiratory and neurological sequelae of COVID-19. Med Sci Monit.

[137] Tawakul AA, Al-Doboke AW, Altayyar SA, Alsulami SA, Alfahmi AM, Nooh RT (2021). Guillain-Barré syndrome in the COVID-19 pandemic. Neurol Int.

[138] Dewanjee S, Vallamkondu J, Kalra RS, Puvvada N, Kandimalla R, Reddy PH (2021). Emerging COVID-19 neurological manifestations: present outlook and potential neurological challenges in COVID-19 pandemic. Mol Neurobiol.

[139] Zito A, Alfonsi E, Franciotta D, Todisco M, Gastaldi M, Cotta Ramusino M (2020). COVID-19 and Guillain-Barré syndrome: a case report and review of literature. Front Neurol.

[140] AlKetbi R, AlNuaimi D, AlMulla M, AlTalai N, Samir M, Kumar N (2020). Acute myelitis as a neurological complication of Covid-19: a case report and MRI findings. Radiol Case Rep.

[141] Bellucci M, Germano F, Grisanti S, Castellano C, Tazza F, Mobilia EM (2022). Case Report: Post-COVID-19 Vaccine Recurrence of Guillain-Barré Syndrome Following an Antecedent Parainfectious COVID-19-Related GBS. Front Immunol.

[142] Keddie S, Pakpoor J, Mousele C, Pipis M, Machado PM, Foster M (2021). Epidemiological and cohort study finds no association between COVID-19 and Guillain-Barré syndrome. Brain.

[143] Palaiodimou L, Stefanou M-I, Katsanos AH, Fragkou PC, Papadopoulou M, Moschovos C (2021). Prevalence, clinical characteristics and outcomes of Guillain−Barré syndrome spectrum associated with COVID-19: a systematic review and meta-analysis. Eur J Neurol.

[144] Chiu NC, Chi H, Tai YL, Peng CC, Tseng CY, Chen CC (2020). Impact of wearing masks, hand hygiene, and social distancing on influenza, enterovirus, and all-cause pneumonia during the coronavirus pandemic: retrospective national epidemiological surveillance study. J Med Internet Res.

[145] Restrepo-Vera JL, Llauradó A, Palasí A, González-Martínez V, Gratacòs M, Salvadó M (2023). Immunological, clinical, and epidemiological features of Guillain-Barré syndrome associated with SARS-CoV-2 Infection. Acta Neurol Scand.

[146] Wang L, et al. Association of COVID-19 with New-Onset Alzheimer's Disease. J Alzheimers Dis. 2022;89(2):411–14. 10.3233/JAD-220717 PMC10361652 35912749

[147] Espíndola OM, Brandão CO, Gomes YCP, Siqueira M, Soares CN, Lima MASD (2021). Cerebrospinal fluid findings in neurological diseases associated with COVID-19 and insights into mechanisms of disease development. Int J Infect Dis.

[148] Hussain FS, Eldeeb MA, Blackmore D, Siddiqi ZA (2020). Guillain Barré syndrome and COVID-19: possible role of the cytokine storm. Autoimmun Rev.

[149] Maxeiner HG, Marion Schneider E, Kurfiss ST, Brettschneider J, Tumani H, Bechter K (2014). Cerebrospinal fluid and serum cytokine profiling to detect immune control of infectious and inflammatory neurological and psychiatric diseases. Cytokine.

[150] Miossec P, Kolls JK (2012). Targeting IL-17 and TH17 cells in chronic inflammation. Nat Rev Drug Discov.

[151] Gupta M, Weaver DF (2021). COVID-19 as a Trigger of Brain Autoimmunity. ACS Chem Neurosci.

[152] Rinaldi V, Bellucci G, Buscarinu MC, Reniè R, Marrone A, Nasello M (2022). CNS inflammatory demyelinating events after COVID-19 vaccines: a case series and systematic review. Front Neurol..

[153] Abdelhady M, Husain MA, Hawas Y, Elazb MA, Mansour LS, Mohamed M (2023). Encephalitis following COVID-19 vaccination: a systematic review. Vaccines.

[154] Toljan K, Amin M, Kunchok A, Ontaneda D (2022). New diagnosis of multiple sclerosis in the setting of mRNA COVID-19 vaccine exposure. J Neuroimmunol.

[155] Tagliaferri AR, Horani G, Stephens K, Michael P (2021). A rare presentation of undiagnosed multiple sclerosis after the COVID-19 vaccine. J Community Hosp Intern Med Perspect.

[156] Mirmosayyeb O, et al. Multiple sclerosis (MS) and neuromyelitis optica spectrum disorder (NMOSD) following COVID-19 vaccines: a systematic review. Rev Neurol (Paris). 2023;179(4):265–81. 10.1016/j.neurol.2022.11.004 PMC9844421 36658048

[157] Czarnowska A, Kapica-Topczewska K, Tarasów E, Tarasiuk J, Chorąży M, Kochanowicz J (2023). Case report: First manifestation of multiple sclerosis temporally correlated with COVID-19 vaccination. Front Neurol.

[158] Stefanou MI, et al. Safety of COVID-19 vaccines in multiple sclerosis: a systematic review and meta-analysis. Mult Scler. 2023;29(4-5):585–94. 10.1177/13524585221150881 PMC9895285 36722184

[159] Yazdani A, Mirmosayyeb O, Ghaffary EM, Hashemi MS, Ghajarzadeh M (2022). COVID-19 vaccines and patients with multiple sclerosis: willingness, unwillingness and hesitancy: a systematic review and meta-analysis. Neurol Sci.

[160] Hussein HM, Rahal EA (2019). The role of viral infections in the development of autoimmune diseases. Crit Rev Microbiol.

[161] Pignolo A, Aprile M, Gagliardo C, Giammanco GM, D'Amelio M, Aridon P (2021). Clinical onset and multiple sclerosis relapse after SARS-CoV-2 infection. Neurol Int.

[162] Jaisankar PJ, Kucera A, Lomiguen CM, Chin J (2021). Complications of COVID-19 pneumonia and multiple sclerosis exacerbation. Cureus.

[163] Barzegar M, Vaheb S, Mirmosayyeb O, Afshari-Safavi A, Nehzat N, Shaygannejad V (2021). Can coronavirus disease 2019 (COVID-19) trigger exacerbation of multiple sclerosis? A retrospective study. Mult Scler Relat Disord.

